# Studies of the symptom dyspnoea: A systematic review

**DOI:** 10.1186/s12875-015-0373-z

**Published:** 2015-10-24

**Authors:** Annika Viniol, Dominik Beidatsch, Thomas Frese, Milena Bergmann, Paula Grevenrath, Laura Schmidt, Sonja Schwarm, Jörg Haasenritter, Stefan Bösner, Annette Becker

**Affiliations:** Department of General Practice / Family Medicine, University of Marburg, Karl-von-Frisch-Str. 4, 35043 Marburg, Germany; Department of General Practice / Family Medicine, University of Leipzig, Ph.-Rosenthal-Str. 55, 04103 Leipzig, Germany

**Keywords:** Dyspnoea, General practice, Primary care, Systematic review, Symptom evaluating study, Prevalence, Aetiology

## Abstract

**Background:**

To deal with patients suffering from dyspnoea, it is crucial for general practitioners to know the prevalences of different diseases causing dyspnoea in the respective area and season, the likelihood of avoidable life-threatening conditions and of worsening or recovery from disease.

**Aim:**

Aim of our project was to conduct a systematic review of symptom-evaluating studies on the prevalence, aetiology, and prognosis of dyspnoea as presented to GPs in a primary care setting.

**Methods:**

We did a systematic review of symptom-evaluating studies on dyspnoea in primary care. For this we included all studies investigating the complaint “dyspnoea” as a primary or secondary consulting reason in general practice. Apart from qualitative studies, all kind of study designs independent from type of data assessment, outcome measurement or study quality were included. Symptom-evaluating studies from other settings than primary care and studies which exclusively included children (age <18 years) were excluded from the review. Studies selecting patients prior to recruitment, e.g. because of an increased probability for a particular diagnosis, were also excluded.

**Results:**

This systematic review identified 6 symptom evaluating studies on dyspnoea in the primary care setting. The prevalence of dyspnoea as reason for consultation ranges from 0.87 to 2.59 % in general practice. Among all dyspnoea patients 2.7 % (CI 2.2–3.3) suffer from pneumonia. Further specification of underlying aetiologies seems difficult due to the studies’ heterogeneity showing a great variety of probabilities.

**Conclusion:**

There is a great lack of empirical evidence on the prevalence, aetiology and prognosis of dyspnoea in general practice. This might yield uncertainty in diagnosis and evaluation of dyspnoea in primary care.

## Background

Dyspnoea is defined as “a subjective experience of breathing discomfort that is comprised of qualitatively distinct sensations that vary in intensity” [1]. The symptom can be caused by a broad spectrum of diseases from mostly trivial and self-limiting (e.g. common cold, somatisation disorder) to acute life-threatening (e.g. pulmonary embolism) ones.

Clinical decision making has to rely on disease distributions and probabilities of underlying aetiologies and the expected course of disease, as well as the nature and pattern of symptoms. To deal adequately with dyspnoea general practitioners need to know the prevalences of different diseases causing dyspnoea in the respective age group, area and season, the likelihood of avoidable life-threatening conditions and of worsening or recovery from disease.

There is empirical evidence of the respective probabilities in the secondary care setting: Mockel et al. described a dyspnoea prevalence of 7.4 % in the emergency departments [2]. Also, data from the U.S. National Hospital Ambulatory Medical Care Survey showed a rate of 8.4 % among 15 to 64 year old emergency patients [3], leading to a considerably high in-hospital mortality of 9.4 % [2].

However, access to primary or secondary care differs, since general practitioners are often the first point of contact, whereas secondary care physicians are consulted by those patients who suffer from more severe symptoms, who do not recover or in whom general practitioners suspect a more severe underlying cause of disease. Prevalences and probabilities from secondary care are not applicable to at the primary care situation. So far there are no evidence-based algorithms or guidelines for the diagnostic work-up of patients presenting with dyspnoea in general practice. Aim of our project was to conduct a systematic review of symptom-evaluating studies on the prevalence, aetiology, and prognosis of dyspnoea (as main or secondary complaint) as presented to general practitioners in daily setting.

## Methods

### Types of studies

This is a systematic review including symptom-evaluating studies about dyspnoea at general practice. According to Donner-Banzhoff et al., symptom evaluating studies are defined as studies examining patients presenting with a defined symptom in health care settings. They aim to investigate prevalence/incidence, differential diagnosis and prognosis for patients presenting with the symptom [4].

### Inclusion and exclusion criteria

We included all studies investigating the complaint “dyspnoea” as a primary or secondary consulting reason at general practice. The findings of the studies had to comprise data about the incidence or prevalence of dyspnoea, a statement about underlying diagnoses and/or prognosis. Apart from qualitative studies, all kind of study designs independent from type of data assessment, outcome measurement or study quality were included. Symptom-evaluating studies from other settings than primary care (primary care is defined as the first-contact care of patients at the health care system which is accessible at the time of need, continued, comprehensive and coordinated [5]; secondary care is offered by medical specialists who usually do not have first contact with patients at the health care system or only temporally in emergency cases) and studies which exclusively included children (age <18 years) were excluded from the review. Studies selecting patients prior to recruitment, e.g. because of an increased probability for a particular diagnosis, were also excluded.

### Search strategy

We did a computer-based search with the PubMed database in May 2012. The following search syntax was used: The term “dyspnoea” in title or abstract OR the MESH term “dyspnea” AND the term “general practice” in title or abstract OR a journal representing our research area OR the term “general practice” (in affiliation of authors) OR the MESH terms “family practice”, “physicians, family” and “primary health care”. All terms were used in various notations. The entire syntax is available on request from the authors (Appendix).

### Selection of publications

All identified references went to a two step-selection process. First, we screened titles and abstracts regarding to the three criteria, “original research article”, “inclusion of patients because of dyspnoea”, and “primary care setting”. References meeting all criteria were classified as potential appropriate. In the second step, we analysed the full texts of the potentially appropriate studies with respect to inclusion- and exclusion criteria. Every step of the selection process was done and documented by two independent review authors (DB, MB). Different appraisals were resolved by discussion between DB und MB. In cases of persisted diversities, a third author (AV) was consulted.

### Data extraction

We extracted the following descriptive data of the included studies: Bibliographic information (author, publication year, title, journal), country, setting, study design, inclusion and exclusion criteria, type of recruitment, study population (age, gender distribution), and study duration. To answer the first research question (prevalence/incidence), we registered the number of patients with the consultation reason dyspnoea, the number and type of the population from which the cases descended from (e.g. number of all practice consultations or all registered patients in a practice). Furthermore, we extracted all diagnostic categories and their absolute and relative frequencies (second question “aetiology”). Finally, every kind of prognostic outcome was documented (third research question).

### Quality assessment

Until now, there is no published standardized and accepted quality or reporting guideline for studies of symptoms. In accordance to Donner-Banzhoff et al. [4], our research group has developed criteria to query essential quality characteristics for studies of symptoms. A validation study is ongoing. Independent of the particular research question, all included studies underwent quality assessment regarding the criteria of Table 4.

### Data analysis

We did the meta-analysis with the random effects model and calculated confidence intervals to show the precision of the mean [6]. Since our review includes studies with various study sizes, we used tau^2^ and I^2^ for quantifying heterogeneity. We approximately estimated the 95 % prediction interval using [expit(PE - 2*tau); expit(PE + 2*tau)], where prediction interval is the random effects pooled estimate of the proportion on the logit scale and expit is the inverse logit function. We used the logit transformation because the included studies of our review mostly contain proportions less than 0.2 or more than 0.8 [7]. The prediction interval describes the distribution of the true effect size of the included studies and is an estimate of an interval in which the true effect size (e.g. prevalence of a symptom) of a future study will fall with a probability of 95 % [8]. While the confidence interval quantifies the uncertainty in the estimation of the true effect size, the prediction interval reflects the between study heterogeneity. The fact that the values of the prediction intervals are equal to the scale of the original results simplifies the clinical interpretation and makes it more ostensive. We did no data pooling in cases where prediction intervals were broader than 10 %.

Data analysis was done with the statistical program R 2.14.0 (R Foundation for statistical analysis, Vienna, Austria). We used the R package “meta: Meta-Analysis with R” [9]. Confidence intervals of frequencies were calculated as exact binomial confidence intervals according to Clopper Pearson.

## Results

### Search results and study selection

We identified 1915 references via the computer-based search in PubMed. After title and abstract screening, 117 studies underwent full text analysis. Thereby, six studies fulfilled the inclusion criteria [10–15]. During the full text analysis, we excluded the following studies for the following reasons: not primary care (26 publications); inadaequate study design/population (five publications); pre-selected study population (ten publications); dyspnoea was not the reason for consultation (52 publications); missing outcome (one publication); full texts not available or other language than English or German (17 studies).

### Included studies

Apart from a study by Charles et al., which was performed in Australia, all studies originate from European countries. The publication time ranges from 2002 to 2012. The studies included patients of every age group, mostly with a slightly female surplus. All studies recruited data at general practices; recruitment duration was 11.5 to 120 months. The study by Charles et al. extracted data out of a database in a retrospective way; all other studies showed prospective patient recruitment. Half of the studies were primarily performed to answer a symptom evaluating research question. In contrast, the studies from Burri and Nielsen et al. mainly evaluated diagnostic tests for diagnostic decision making of dyspnoea while the symptom evaluation data derive from a secondary analysis. Further details of the included studies are shown in Table 1.

**Table 1:** Brief description of the included studies

Studies	Recruitment duration [month]	Region	Setting	Age distribution of study sample	Female (%)	Data assessment	Inclusion criteria	Exclusion criteria	Answered research questions
Burri, 2012	---	Germany + Switzerland	29 primary care physicians	Median age: 72 years	53.9	prospective	- all patients presented with dyspnoea as their primary symptom	- < 18 years	2^b^ + 3^c^
								- obvious traumatic cause of dyspnoea	
							- Dyspnoea had to be of new onset or clearly worsening if preexisting	- severe renal disease [defined by a serum	
								creatinine level of more than 250 μmol /L^−1^ (2.8 mg/dL^−1^)	
								- sepsis	
Charles, 2005 (BEACH Program)	72	Australien	6021 general practitioners	<5 years: 3.0 %	53.2	retrospective	- all documented patients with shortness of breath	---	1^a^ + 2^b^
				5–14 years: 3.3 %					
				15–24 years: 4.6 %					
				25–44 years:10.8 %					
				45–64 years: 21.2 %					
				65–74 years: 21.7 %					
				75+ years: 35.3 %					
Frese, 2011 (SESAM Study)	12	Germany	270 general practitioners	Mean age: 51.2 years, SD +/−20.86	56.9	prospective	- all patients with a direct (face to face) GP contact; independent from consultation reason	none	1^a^ + 2^b^
				Median age: 55 years					
Nielsen, 2001	11.5	Denmark	74 general practitioners	Mean age: 63.0 years	47.7	prospective	- all patients with dyspnoea of at least 2 weeks duration	- patients with dyspnoea of at least less 2 weeks duration	2^b^ + 3^c^
Nielsen, 2003	24	Denmark	74 general practitioners	Median age: 65.0 years	49.0	prospective	- all patients with dyspnoea of at least 2 weeks duration	- patients with dyspnoea of at least less 2 weeks duration	2^b^
Okkes, 2002	12–120 (mean: 2.4 years)	Netherlands	54 family physicians	---	56.5	prospective	- all patients with a direct (face to face) GP contact; independent from consultation reason	none	1^a^ + 2^b^
Frese, 2011 (Transition Project^d^)									

^a^First research question: Prevalence of the consulting reason dyspnoea at general practice

^b^Second research question: Aetiology of the consulting reason dyspnoea at general practice

^c^Third research question: Prognosis of the consulting reason dyspnoea at general practice

^d^Both studies from Frese and Okkes published data from the Transitions Project persist. Due to the more detailed data presentation, we extracted the data from the article from Frese et al

### Prevalence of “dyspnoea” in general practice

We extracted prevalence data out of three studies referring to 9 051 dyspnoea cases and 760 215 consultations. The prevalence of dyspnoea in general practice ranges from 0.87 to 2.59 % (Table 2). The database study by Charles et al. reports a prevalence of 0.87 % (CI 0.85–0.89) and the non-database studies from Frese and Okkes et al. found a prevalence of 1.05 % (CI 0.85–1.29) and 2.59 % (2.43–.2.59). Due to the low number of studies, we performed no a meta-analysis. Please see Table 2 for further details.

**Table 2:** Prevalence

	Number of patients with the consultation reason dyspnoea^a^	Overall consultations	Prevalence	CI
Charles, 2005 (BEACH Program)	5215	602100 ^b^	0.87 %	0.85–0.89
Frese, 2011 (SESAM Study)	93	8877 ^b^	1.05 %	0.85–1.29
Okkes, 2002	3743	149238 ^c^	2.59 %	2.43–2.59
Frese, 2011 (Transition Project ^d^)				

^a^Patients with several consultations were singular counted during assessment time

^b^All direct encounters during assessment time (without double consultations)

^c^All active listed patients

^d^Both studies from Frese and Okkes published data from the Transitions Project persist. Due to the more detailed data presentation, we extracted the data from the article from Frese et al

### Aetiologies of “dyspnoea” in general practice

All six included studies provided data about the underlying aetiologies of dyspnoea. The authors described a broad spectrum of differential diagnoses which were summarized in twelve categories, see Table 3. The presented diagnoses show a high heterogeneity which was confirmed by both heterogeneity sizes (I^2^ and tau^2^). Therefore we omitted meta-analysis. Merely the analysis of the diagnostic category “pneumonia” turns out with high homogeneity (I^2^ and tau^2^ = 0) enabling meta-analysis: The probability for pneumonia as an underlying reason for dyspnoea at primary care setting is 2.7 % (CI 2.2–3.3).

**Table 3:** Aetiologies of the symptom “dyspnoea” in general practice

N	Burri et al. 323	Charles et al. 5200	Frese et al. 93	Nielsen (2001) et al. 284	Nielsen et al. (2004) 345	Okkes et al.^f^ Frese et al.^f^ (Transition Project) 3743	Prädiktions-intervall [%]	Tau^2^	I^2^	P value	Random effects model
Simple respiratory infect (mostly viral)	---	587	37	---	---	1498	3.7–79.0	1.3146	99.8 %	<0.0001	---
		11.3 %	39.8 %			40.1 %					
		10.5–12.2	29.9–50.5			38.5–41.6					
Pneumonia	11	---	3	---	---	99	2.7–2.7	0	0 %	0.6907	2.7 [2.2–3.3]
	3.4 %		3.2 %			2.6 %					
	1.8–6.2		0.8–9.8			2.2–3.2					
COPD / chronic bronchitis	94	998	9	---	---	112	1.3–58.6	1.3833	99.3 %	<0.0001	---
	29.1 %	19.2 %	9.7 %			3.0 %					
	24.3–34.4	18.1–20.3	4.8–18.0			2.5–3.6					
Asthma / Allergy	---	1092	5	---	---	431	4.9–28.6	0.2632	98.6 %	<0.0001	---
		21.0 %	5.4 %			11.5 %					
		19.9–22.1	2.0–12.7			10.5–12.6					
Other pulmonary diseases (neoplasia, pulmonary embolism)	---	---	---	---	---	18	---	---	---	---	---
						0.5 %					
						0.3–0.8					
Heart failure	115	946	6	---	---	153	1.6–54.8	1.1580	99.3 %	<0.0001	---
	35.6 %	18.2 %	6.5 %			4.1 %					
	30.4–41.1	17.2–19.3	2.7–14.1			3.5–4.8					
Other cardiovascular diseases	---	650	9	---	20	79	0.6–40.6	1.3523	98.8 %	<0.0001	---
		12.5 %	9.7 %		5.8 %	2.1 %					
		11.6-13.4	4.8–18.0		3.7–9.0	1.7–2.6					
Psychosomatic cause	13	177	1	---	2	314	0.9–12.3	0.4524	96.5 %	<0.0001	---
	4.0 %	3.4 %	1.1 %		0.1 %	8.4 %					
	2.3-7.0	2.9–3.9	0.06–6.7		0.1–2.3	7.5–9.3					
Musculoskeletal cause	---	---	2	---	---	3	0.0–26.9	5.0575	92.3 %	0.0003	---
			2.2 %			0.1 %					
			0.4-8.3			0.02–0.3					
Obesity Lack of fitness	29	---	1	---	31	5	0.1–31.9	2.3909	96.6 %	<0.0001	---
	9.0 %		1.1 %		9.0 %	0.1 %					
	6.2-12.8		0.06–6.7		6.3–12.6	0.05–0.3					
Anaemia / metabolism	---	---	---	---	4	8	0.1–4.3	1.2567	86.9 %	0.0057	---
					1.2 %	0.2 %					
					0.4-3.2	0.1–0.4					
No diagnosis	61	447	12	42	62	529	7.0–26.3	0.1503	95.0 %	<0.0001	---
	18.9 %	8.6 %	12.9 %	14.8 %	18.0 %	14.1 %					
	14.9-23.7	7.9–9.4	7.1–21.8	11.0–19.6	14.2–22.5	13.0–15.3					
Other	---	a	b	c	d	e					

a-Diagnoses in 303 cases (5.8 %, CI 5.2–6.5) are not listed at article

b-Sleep disorder: 1 (1.1 %, CI 0.06–6.7)

-Prevention/no disease 2 (2.2 %, CI 0.4–8.3)

-Diagnoses in 5 cases (5.4 %, CI 2.0–12.7) are not listed at article

c-Heart failure (systolic dysfunction, diastolic dysfunction, atrial fibrillation, valvular disease, secondary pulmonary hypertension): 48 (16.9 %, CI 12.8–21.9)

-Lung disease (COPD, asthma, α-1 antitrypsin deficiency, restrictive lung disease, thoracic deformities, lung cancer, stenosis trachea): 100 (35.2 %, CI 29.7–41.1)

-Combined heart and lung disease:40 (14.1 %, CI 10.4–18.8)

-Other well defined reason: 54 (14.1 %, CI 10.4–18.8)

d-Heart failure (systolic dysfunction, diastolic dysfunction, atrial fibrillation, valvular disease, secondary pulmonary hypertension): 51 (14.8 %, CI 11.3–19.1)

-Lung disease (COPD, asthma, α-1 antitrypsin deficiency, restrictive lung disease, thoracic deformities, lung cancer, stenosis trachea): 136 (39.4 %, CI 34.3–44.8)

-Combined heart and lung disease: 30 (8.7 % CI 6.1–12.3)

-Angina pectoris: 20 (5.8 CI 3.7–4.0)

-Neurologic origin: 2 (0.6 % CI 0.1–2.3)

-Hypertension: 2 (0.6 % CI 0.1–2.3)

-Paroxysmal tachycardia: 1 (0.3, CI 0.02–1.9)

-Intrathoracic goiter: 1 (0.3, CI 0.02–1.9)

-Allergy: 1 (0.3, CI 0.02–1.9)

-Side effect from medication: 1 (0.3, CI 0.02–1.9)

-Malignant disease:1 (0.3, CI 0.02–1.9)

e-Prevention/no disease 56 (1.5 %, CI 1.2–2.0)

-Diagnoses in 441 cases (11.8 %, CI 10.8–12.9) are not listed at article

^f^Both studies from Frese and Okkes published data from the Transitions Project persist. Due to the more detailed data presentation, we extracted the data from the article from Frese et al

### Prognosis of “dyspnoea” in general practice

Two studies reported prognostic outcome parameters. Burri et al. documented 94 hospitalizations and 20 case of death among 323 patient during a one year follow-up [10]. In addition, a symptom evaluation after three month was done showing 32 % of the respective patients to be symptom free [10]. Nielsen et al. assessed the mortality rates and the symptom status among 269 dyspnoea patients during a six month follow-up [13]: 58 % of the patients reported symptom improvement, 34 % experienced no change and 5 % showed worsening; 3 % were dead (all reasons).

### Quality of included studies

Regarding to domain A (selection of patients and GPs), risk of bias of most included studies (5/6) was classified as unclear, because quality assessment criteria regarding to selection of patients and GPs were not adequately described. In the study of Burri et al., the risk that the selection of patients introduced bias was judged as low. All included studies showed a low risk that the mode of data collection and/or patients flow introduced bias (domain B). Quality assessment for the determination of the underlying aetiology of the symptom (domain C) was done for every diagnostic category of each study. The two studies from Nielsen et al. had the lowest risk that the diagnostic work up introduce bias. In contrast, the three studies from Charles et al. Frese et al. and Okkes et al. showed a predominantly high risk that the diagnostic work up introduce bias. Regarding to the determination of prognosis (domain D), the two studies containing prognostic information contain a high risk for bias. The detailed results of the quality assessment are described at Table 4.

**Table 4:** Methodical quality of the included studies

	Burri et al.	Charles et al.	Frese et al.	Nielsen et al. (2001)	Nielsen et al. (2004)	Okkes et al.
*Domain A: Selection of patients and GPs (refers to all studies regardless the review question)*						
Was the symptom to be investigated clearly described?	no	no	no	no	no	no
Were the selection criteria of the patients clearly described?	yes	yes	yes	unclear	unclear	yes
Was a consecutive or random sample of patients enrolled?	yes	yes	yes	yes	yes	yes
Was it a multi-centre study?	yes	yes	yes	yes	yes	yes
Did the selection criteria of the patients permit the study population to represent the full spectrum of those presenting with the symptom in the respective setting/ addressed in the review question?	yes	unclear	unclear	yes	no	unclear
Were the participating health care professionals/ institutions representative for setting to be investigated in the review?	yes	yes	yes	yes	yes	yes
**Concern that the selection of patients and GPs introduced substantial variation**	*low*	*low*	*low*	*unclear*	*unclear*	*low*
**Risk that the selection of patients introduced bias: low, unclear, high**	*low*	*unclear*	*unclear*	*unclear*	*unclear*	*unclear*
*Domain B: Data collection and patient flow (refers to all studies regardless of the review question)*						
Were data about the symptom und the inclusion criteria collected directly from the patients (as opposed to a proxy like a register, routine documentation)?	yes	yes	yes	yes	yes	yes
Was the same mode of data collection used for all patients?	unclear	yes	yes	yes	yes	yes
Was the number of non-responders/ dropouts unlikely to affect the results?	yes	unclear	unclear	yes	yes	unclear
**Risk that the mode of data collection and/ or patient flow introduced bias: low, unclear, high**	*low*	*low*	*low*	*low*	*low*	*low*
*Domain C: Determination of the underlying aetiology of the symptom (refers only to review question “What are the underlying conditions and their respective frequencies (differential diagnosis)?”)*						
Was the etiologic category clearly defined?	Yes (3/6) No (2/6) ^a^ (1/6)	Yes (5/7) No (1/7) ^a^ (1/7)	Yes (9/10) ^a^ (1/10)	Yes (4/6) No (1/6) ^a^ (1/6)	Yes (4/6) Unclear (1/6) ^a^ (1/6)	Yes (11/12) ^a^ (1/12)
Was the diagnostic work up likely to correctly classify the respective aetiology?	Yes (5/6) ^a^ (1/6)	Yes (6/7) ^a^ (1/7)	Yes (9/10) ^a^ (1/10)	Yes (3/6) Unclear (2/6) ^a^ (1/6)	Yes (4/6) Unclear (1/6) ^a^ (1/6)	Yes (11/12) ^a^ (1/12)
Did every patient receive the same diagnostic work up to detect the respective aetiology?	No (5/6) ^a^ (1/6)	No (6/7) ^a^ (1/7)	Yes (9/10) ^a^ (1/10)	Yes (3/6) No (1/6) Unclear (1/6) ^a^ (1/6)	Yes (3/6) No (2/6) ^a^ (1/6)	No (11/12) ^a^ (1/12)
**Risk that the diagnostic work up introduce bias**	*low (2/6)*	*high (6/7)*	*high (9/10)*	*low (3/6)*	*low (4/6)*	*High (11/12)*
	*unclear (2/6)*	^*a*^ *(1/7)*	^*a*^ *(1/10)*	*Unclear (2/6)*	*unclear (1/6)*	^*a*^ *(1/12)*
	^*a*^ *(1/6)*			^*a*^ *(1/6)*	^*a*^ *(1/6)*	
*Domain D: Determination of the prognosis (refers only to review question “What is the prognosis of patients with the respective symptom presenting in the respective setting?”)*						
Was the prognostic outcome clearly defined?	Yes (3/3)	-	-	Yes (2/2)	-	-
Did the study design include a comparison group without the symptom?	No (3/3)	-	-	No (2/2)	-	-
Was the work up/ measurement likely to correctly classify the respective prognostic outcome?	Yes (3/3)	-	-	Yes (2/2)	-	-
Did every patient receive the same work up/ mode of data collection to verify the respective prognostic outcome?	Yes (3/3)	-	-	Yes (2/2)	-	-
**Risk that the prognostic work up introduce bias**	*High (3/3)*	*-*	*-*	*High (2/2)*	*-*	*-*

^a^The diagnostic category “no diagnosis” were not judged

## Discussion

### Main findings

This systematic review identified 6 symptom evaluating studies on dyspnoea in the primary care setting. The prevalence of dyspnoea as reason for consultation ranges from 0.87 to 2.59 % in general practice. Among all dyspnoea patients 2.7 % (CI 2.2–3.3) suffer from pneumonia. Further specification of underlying aetiologies seems difficult due to the studies heterogeneity showing a great variety of probabilities.

### Interpretation of findings in relation to previously published work

In contrast to secondary care setting (dyspnoea prevalence at emergency departments: 7.4 % [2]), we found rather low prevalences of dyspnoea ranging from 0.87 to 2.59 % at primary care setting. The Transitions Project, provided in the article from Frese et al., showed a higher prevalence value compared to the two other studies. This can be explained by the different formations of the underlying study populations (denominator of the prevalence ratio). At BEACH- and SESAM project, the prevalence ratio was calculated by division of the number of dyspnoea patients (counter) through all direct encounters during assessment time (without double consultations) (denominator). In contrast, at Transition project the prevalence value was calculated by the number of dyspnoea patients (counter) through all active listed patients (denominator).

With exception of the diagnostic category “pneumonia”, the results of the different studies show high heterogeneity. The study by Charles et al. seems to differ from all other studies with respect to the distribution of underlying aetiologies [11]. Again this possibly refers to the chosen study design. Charles’s study refers to a retrospective primary care register. Possibly in this underlying chronic diseases of dyspnea were documented more frequently than acute illnesses (e.g. a simple respiratory infection), because documentation of chronic diseases are more relevant concerning long term care and possibly reimbursement. In contrast, the prospective studies from Okkes and Frese showed a higher rate of respiratory infections [12, 15], where recruitment of dyspnea patients was done in a consecutive way.

Furthermore, there are large differences between the studies regarding the diagnostic categories “COPD/chronic bronchitis”, “asthma/allergy” and “heart failure”. This is most likely based on the different age distribution of the study populations and different diagnostic strategies. In fact the study by Burri et al., had a special focus on the diagnosis of heart failure and the average age of the studies’ population was higher compared to the other studies’ participants [10].

All studies of the review have included patients with dyspnea independent from duration and severity of the symptom which might result in differences in the proportionately composition between acute and chronic dyspnoea cases among the study populations. Even though we have no evidence from this review one would expect that chronic dyspnea is mainly related to diseases like heart failure, chronic bronchitis etc. whereas acute dyspnoea is most likely associated with pneumonia, viral infections or pulmonary embolism. This might be even add to the different distribution of the diagnostic categories.

In general, most diagnoses analyzed in the included studies are based on clinical non standardized criteria with great variety between studies. This leads to substantial blurring in the estimated probabilities. In comparison to other symptoms (like abdominal pain or dizziness), the proportion of unexplained complaints (category “no diagnosis”) is rather rare among dyspnea patients [16, 17]. GPs seem to have hypotheses concerning the underlying aetiology; although only 39 % of these turn out to be correct [13]. Nielsen et al. showed improvement of GP’s aetiological appraisal when providing diagnostic work up in a hospital. Nielsen reported that GPs tend to overdiagnose heart failure (overdiagnosed: 63 %; missed: 39 %). Diagnosis of pulmonary disease showed equipoise of overdiagnosis and missing (overdiagnosed: 50 %; missed 57 %) [13]. At present, there are no primary care decision rules for the evaluation of dyspnea.

### Strengths and limitations of this study

Systematic reviews of symptom-evaluating studies underlie four factors which could bias the effect size of this systematic review from the real effect size [18]: (i) Factors which influence the internal validity of the included studies, like incomplete recruitment or imprecise inclusion criteria; (ii) Factors which might influence the external validity of the included studies, like setting or recruitment characteristics which impede transferability to the local health care system; (iii) Factors which influence the internal validity of our systematic review based on our own methodology; (iiii) Factors influencing the external validity of the review. We therefore followed a transparent and standardized protocol for the quality assessment of the included studies. The screening process was done by two independent reviewers and we defined clear inclusion criteria for the included studies.

There are few publications which describe quality criteria for prevalence studies [19, 20]: Hoy et al. defined a 10-item risk of bias tool for prevalence studies and tested its interrater-reliability [19] and the Evidence-Based Medicine Working Group from Richardson et al. developed criteria to evaluate articles on disease probability for differential diagnosis [20]. These published criteria only comment on the quality of prevalence studies. We developed a more comprehensive catalogue of criteria based on an extensive literature review and the Standards for the Reporting of Diagnostic Accuracy (STARD) statement on diagnostic accuracy studies [18, 21]. According to our quality criteria, quality of the included studies shows a broad spectrum from low to high risk of bias.

A search update (28/08/2015) identified 649 new references since May 2012. After title and abstract screening, 12 studies underwent full text analysis. Finally, one study fulfilled the inclusion criteria [22]. This study from Currow et al. was based on the same database (BEACH Programm) like the already included publication from Charles et al. and answered the first (prevalence) and the second (aetiology) research question. In comparison to analysis from Charles at al., Currow et al. covered a longer recruitment period (Charles: 6 years vs. Currow: 9 years) and excluded persons under 18 years. Currow found a prevalence of 0.96 % (95 %-CI: 0.93–0.99). The following percentage distributions of the underlying aetiologies were described: chronic obstructive pulmonary disease (10.4 %), asthma (9.6 %), heart failure (9.4 %), hypertension (4.1 %), acute bronchitis/bronchiolitis (4.0 %), ischaemic heart disease (3.0 %), sleep disturbance (2.4 %) and anxiety (2.1 %). In summary, the results of the two publications are generally similar; the existing differences are explainable due to the different age-sample.

### Implication for future research, policy and practice

Although, GPs need setting specific knowledge about the pre-test probability (prevalence), the work-up probability (suspected underlying aetiology), and the impact of diagnostic testing (mainly generated from patient history and clinical examination) in dyspnoea, there are only few symptom evaluating studies published to support their decision making. A comprehensive diagnostic study with sound methodology regarding recruitment, standardized diagnosis, and follow up is needed in order to gain empirical data for future guidelines and decision rules.

## Conclusion

There is a great lack of empirical evidence on the prevalence, aetiology and prognosis of dyspnoea in general practice. This might yield uncertainty in diagnosis and evaluation of dyspnoea in primary care.

### Ethical approval

A systematic review does not require ethical approval.

## Appendix

**Table 5:** Search syntax for PubMed

term “dyspnoea” in various notations (in title)	(dyspn* [title] OR (short* [title] AND breath* [title]) OR SOB [title] OR (laboured [title] AND breath* [title]) OR (labored [title] AND breath* [title]) OR (laboured [title] AND respirat* [title]) OR (labored [title] AND respirat* [title]) OR (short* [title] AND wind* [title]) OR breathless* [title] OR (difficult [title] AND respirat* [title])
MESH term “dyspnoea”	dyspnea [Mesh]
term “general practice” in various notations (in title or abstract)	(“general practi*” [TIAB] OR “family practi*” [TIAB] OR “family medicine” [TIAB] OR “family physician” [TIAB] OR “family doctor*” [TIAB] OR “primary care” [TIAB])
journal representing our research area	(“BMC Fam Pract”[TA] OR “Fam Pract”[TA] OR “J Fam Pract”[TA] OR “Fam Pract Res J”[TA] OR “J Am Board Fam Pract”[TA] OR “Br j gen pract”[TA] OR “Can fam physician”[TA] OR “Ann Fam Med”[TA] OR “Aust fam physician”[TA] OR “Scand J Prim Health Care”[TA] OR “Eur J Gen Pract”[TA] OR “Archives of family medicine”[Journal]”)
term “general practice” in various notations (in affiliation to authors)	(“general practice” [AD] OR “family practice*” [AD] OR “family medicine” [AD] OR “primary care” [AD] OR community [AD])
MESH terms “family practice”, “physicians, family” and “primary health care”	(“Family Practice”[Mesh] OR “Physicians, Family”[Mesh] OR “Primary Health Care”[Mesh])

Limits: Editorial, Meta-Analysis, Practice Guideline, Review, Addresses, Bibliography, Biography, Case Reports, Comment, Dictionary, Directory, Festschrift, Government Publications, Guideline, Historical Article, In Vitro, Interactive Tutorial, Interview, Introductory Journal Article, Lectures, Legal Cases, Legislation, News, Patient Education Handout, Portraits, Webcasts
